# Psychological Distress During COVID-19 Curfews and Social Distancing in Saudi Arabia: A Cross-Sectional Study

**DOI:** 10.3389/fpubh.2021.792533

**Published:** 2022-01-04

**Authors:** Badrah S. Alghamdi, Yasser Alatawi, Fahad S. Alshehri, Haythum O. Tayeb, Hanin AboTaleb, Amal Binsalman

**Affiliations:** ^1^Department of Physiology, Faculty of Medicine, King Abdulaziz University, Jeddah, Saudi Arabia; ^2^Pre-Clinical Research Unit, King Fahad Medical Research Center, King Abdulaziz University, Jeddah, Saudi Arabia; ^3^Department of Pharmacy Practice, Faculty of Pharmacy, University of Tabuk, Tabuk, Saudi Arabia; ^4^Department of Pharmacology and Toxicology, College of Pharmacy, Umm Al-Qura University, Makkah, Saudi Arabia; ^5^Division of Neurology, Department of Internal Medicine, Faculty of Medicine, King Abdulaziz University, Jeddah, Saudi Arabia

**Keywords:** mental health, public health, COVID-19, psychological distress, Saudi Arabia

## Abstract

**Background:** Coronavirus disease 2019 (COVID-19) has spread to over 150 countries worldwide. Since the first case of COVID-19 was confirmed in Saudi Arabia, cases have continued to escalate exponentially. The COVID-19 outbreak has had a negative effect on mental health and well-being. The study aimed to investigate the effects of the strict national regulations associated with the COVID-19 pandemic on mental health.

**Methods:** This was a cross-sectional study of a convenience sample of Saudi residents. Saudi residents aged 18 years or older were invited to complete an online questionnaire after one month of a nationwide 24-h curfew between May 6, 2020 and May 13, 2020. We measured psychological distress using the Depression, Anxiety, and Stress Scale-21 (DASS-21). We ran binary logistic regression analyses to detect variables that significantly predicted DASS-21 scores.

**Results:** A sample of 2252 participants was recruited from the general population of Saudi Arabia. The DASS-21 score means and standard deviations for depression and anxiety for the whole sample (10.73 ± 10.29 and 6.98 ± 8.30, respectively) were in the range of mild depression and anxiety. In contrast, the mean DASS-21 stress score was within the normal range (11.97 ± 10.80). The mean stress score for healthcare workers was within the normal range (13.70 ± 10.68) but was significantly higher than the mean score for the public (11.56 ± 10.89; *P* = 0.0006). Several variables (e.g., age, gender, and history of contact with confirmed COVID-19 cases) were significantly associated with higher DASS-21 scores.

**Conclusions:** The COVID-19 pandemic has created a psychological burden. Therefore, there is an urgent need to implement emergency public health interventions that ameliorate the risk perception of COVID-19 through the dissemination of adequate and targeted health information that could be a successful measure to mitigate the psychological impact of the Covid-19 pandemic.

## Introduction

Coronavirus disease 2019 (COVID-19), caused by severe acute respiratory distress syndrome coronavirus 2 (SARS-CoV-2), has developed into a worldwide pandemic since December 2019 (1). As of May 2020, over 4.7 million people have been infected worldwide and there have been more than 300,000 deaths (2). The pandemic has had substantial global health, social and economic effects and resulted in large-scale enforcement of curfew regulations (3, 4).

Pandemics are associated with a significant mental health burden (5–8). Studies demonstrating the mental health impact of COVID-19 have accumulated over recent months. Chinese studies have shown that ~35–50% of people have experienced psychological distress owing to the COVID-19 pandemic (9, 10). Additionally, a multinational study has shown that 26.7% of healthcare workers experienced anxiety symptoms during the outbreaks (11). High rates of psychological distress have also been reported in Australia (12), Italy (13), Mexico (14), the UK (15), France (16), Germany (17), Portugal (18), Brazil (19), Japan (20), Nepal (21), and Iran (22). The mental health burden includes stress, anxiety, depression, post-traumatic stress disorder (PTSD), and insomnia. Several of the above studies indicate that younger age, pre-existing mental health difficulties, and chronic conditions are risk factors of psychological morbidity during pandemics. Because many variables may predispose individuals to psychological distress during pandemics, an increase in health-related anxiety is expected during these periods (23, 24). Disruption to daily economic and social activities as a result of social distancing practices and government lockdown regulations is also associated with substantial distress during pandemics (25). The relationship between the aggressiveness of government lockdown regulations and anxiety has not been sufficiently studied. However, the effects of such regulations on mental health may likely vary depending on the sociodemographic and psychosocial characteristics of the population being studied.

The Kingdom of Saudi Arabia (KSA) provides a model for a systematic, aggressive, nationwide plan to combat pandemics. The government dealt with the COVID-19 pandemic decisively and swiftly. COVID-19 reached the KSA on March 2, 2020, when the first cases were recorded. By March 9, schools were closed, government services scaled-down, and travel restrictions imposed. A full curfew was first imposed on some cities on March 23, 2020 and was then enforced nationwide on April 6, 2020. Public prayers in mosques were suspended. In 2012, the KSA experienced an outbreak of another coronavirus, the Middle East respiratory syndrome coronavirus (MERS-CoV), which may have primed the country and increased the responsiveness of the authorities (26). The potential mental health burden related to COVID-19 in the KSA has not been fully quantified. Although strict restrictions on social and economic activities and travel may cause heightened psychological distress, trust in the authorities' efforts and the potential success of these efforts may mitigate the risk of an increased psychological burden (27). Mental health data from the Saudi setting could provide helpful insights into the determinants of psychological health during pandemics and contribute to comparative studies across countries. Therefore, in this observational cross-sectional study, we aimed to measure the levels of stress, anxiety, and depression experienced by a sample of the public during the strict regulations associated with the COVID-19 pandemic regulations in the KSA.

## Materials and Methods

### Design and Sample

**Figure 1:**
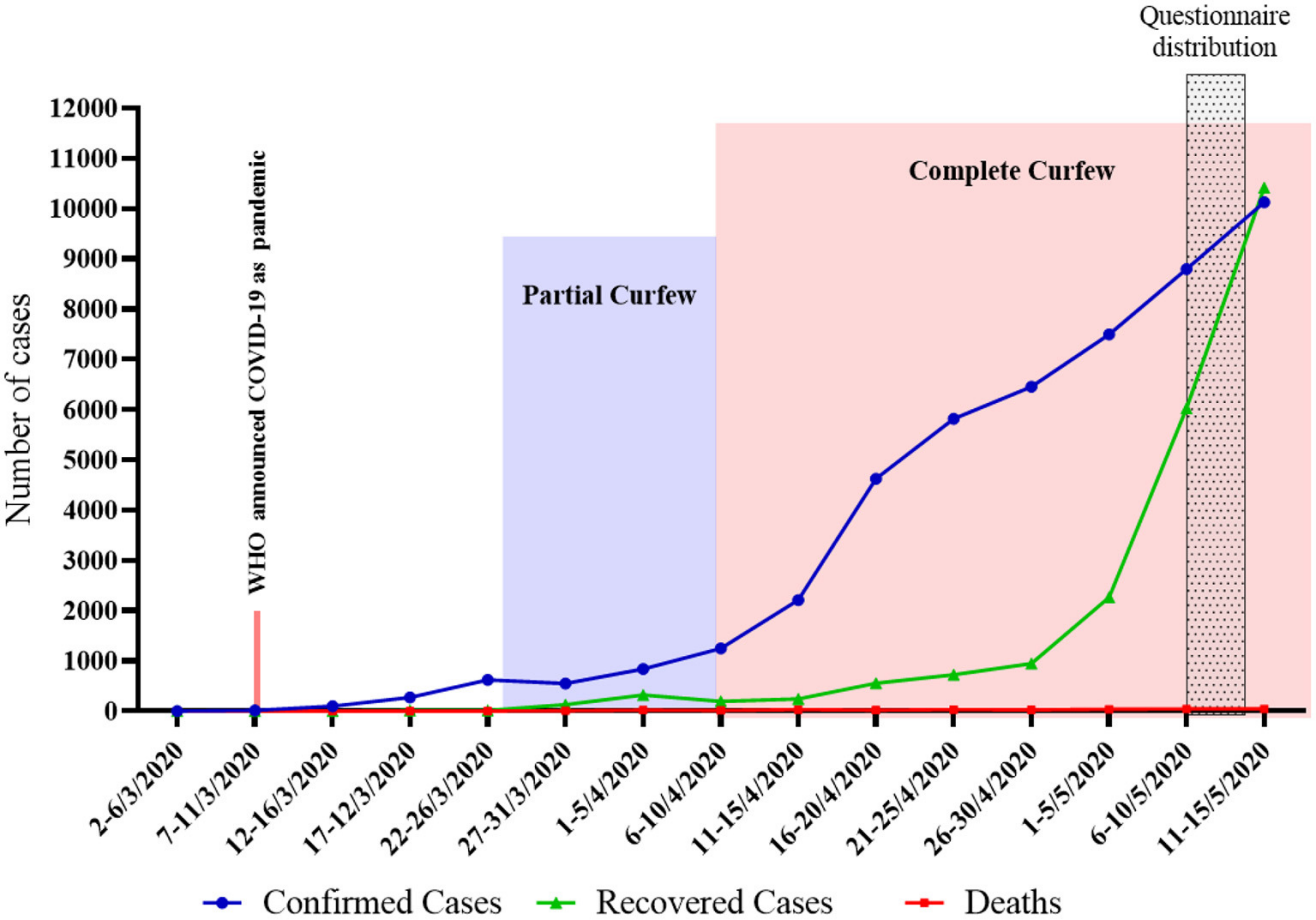
Number of COVID-19 cases in KSA: March 2 (first confirmed case) to May 15 2020. https://covid19.cdc.gov.sa/ar/daily-updates-ar/.

This study was approved by the research ethics committee of King Abdulaziz University (approval no. 234-20). We recruited a convenience sample of Saudi public citizens and residents aged 18 years or older. Web-based digital data collection has been endorsed as an effective way to gain insights into individuals' physical and psychological well-being during pandemics (28). Therefore, given the travel restrictions and enforcement of social distancing, the sample was recruited from the Internet by distributing the study questionnaire on social media platforms and institutional email services. The questionnaire was prepared using one of the author's institutional accounts in Google Forms, a secure online data collection survey tool that allows participants to answer questions conveniently and anonymously. The questionnaire was distributed between May 6 and May 13, 2020, after a month of a nationwide 24-h curfew (20 1) (Figure 1). Participants were instructed to fill the survey once.

### Sociodemographic and DASS-21 Questionnaire

The survey was disseminated in both English and Arabic to facilitate the participation of individuals skilled in both languages. The survey consisted of two sections. The first section contained questions about sociodemographic variables (age, education, marital status, employment status, income, nationality, and Saudi region of residence). We also asked if participants had been diagnosed with COVID-19, if they were healthcare workers and if they had been in contact with a person who had COVID-19. In addition, we inquired whether participants worked as security personnel (e.g., police), as these individuals are responsible for carrying out and monitoring curfew policies in the streets and may therefore be subjected to unique levels of stress.

The second section of the questionnaire contained the Depression, Anxiety, and Stress Scale-21 (DASS-21) (29). The DASS-21 is a simple and validated tool to assess psychological distress in the clinical setting and the community (26, 30). It consists of 21 questions, seven questions for each of the three target mental health phenomena (depression, anxiety, and stress). The scale provides a cutoff value for each subscale. Participants who score above these cutoff values are considered to show mild, moderate, severe, or extremely severe symptoms (30). The total DASS-21 score is also meaningful and denotes the presence of substantial psychological distress. Previous studies have demonstrated the validity of the DASS-21 compared with clinical psychiatric interviews in screening for depression, anxiety, and stress with reasonable sensitivity and specificity (31, 32). The Arabic version of the DASS-21 has been used in published studies (33–35). The DASS-21 has recently shown meaningful results in several studies in other countries assessing mental health in the context of the COVID-19 pandemic (22, 36).

### Sample Size

We used Epi info® version 7 to estimate the study sample. The study sample was estimated to be 1651, assuming that 22.1% of the population has psychological distress symptoms (37). The confidence level was set at 95% and the margin of error at 2%.

### Statistical Analysis

We used frequencies, percentages, means, and standard deviations for descriptive statistics. We calculated the total and subscale DASS-21 scores and subsequently calculated those scores' means and standard deviations. We used a one-way analysis of variance test and chi-square to search for differences in DASS-21 subscale scores (depression, anxiety, and stress) between participant subgroups of interest (the public, healthcare workers, security personnel), given that healthcare and security personnel may theoretically be subjected to more stress regarding COVID-19. Subsequently, we performed logistic and linear regression analysis to identify risk factors for psychological distress and determine their role in the variability of the DASS-21 subscores. The binary outcomes were coded as “abnormal” for DASS-21 subscores above the established clinical thresholds and as “normal” for scores below those thresholds. We calculated the odds ratios (OR) and 95% confidence intervals (CI) based on the probability of having abnormal DASS-21 subscores. We set the threshold for statistical significance at *P* < 0.005 to minimize false positives. We performed statistical analysis using SAS software, version 9.4 (SAS Institute Inc., Cary, NC, USA).

## Results

We collected a total of 2,334 survey responses. We excluded 64 responses from participants younger than 18 years. Another 18 responses were excluded owing to a discrepancy in the response data. We then analyzed data for the remaining 2,252 responses. The sociodemographic characteristics of all groups are presented in Table 1. Most participants were female (65%). Most (60%) were ≤ 38 years; only slightly more than 8% were ≥59 years. Nearly 80% of the sample had a Bachelor's degree or higher. A fifth of our participants were unemployed. Most participants (64%) resided in the western region of the KSA. Only 10% were non-Saudi. Healthcare workers and security force personnel represented (19%) and (4.8%) of the total sample, respectively. Only 2% of the sample had been diagnosed with COVID-19.

**Table 1:** Sociodemographic characteristics of the study sample.

**Variables**		**Total sample**	**Public**	**Healthcare workers**	**Security forces**
		***N* (%)**	***N* (%)**	***N* (%)**	***N* (%)**
**Age (years)**					
18–28		797 (35.39)	597 (35.01)	173 (39.59)	27 (24.55)
29–38		551 (24.47)	377 (22.11)	116 (26.54)	58 (52.73)
39–48		402 (17.85)	327 (19.18)	63 (14.42)	12 (10.91)
49–58		311 (13.81)	246 (14.43)	54 (12.36)	11 (10.00)
>59		191 (8.48)	158 (9.26)	31 (7.09)	2 (1.82)
**Gender**					
Male		792 (35.17)	550 (32.26)	193 (44.16)	49 (44.55)
Female		1460 (64.83)	1155 (67.74)	244 (55.84)	61 (55.45)
**Educational level**					
Less than high school		48 (2.13)	43 (2.52)	2 (0.64)	3 (2.73)
High school		415 (18.43)	365 (21.41)	34 (7.78)	16 (14.55)
Bachelor's degree		1,416 (62.88)	1,064 (62.40)	274 (62.70)	78 (70.91)
Master's degree		214 (9.50)	147 (8.62)	55 (12.59)	12 (10.91)
Doctorate		159 (7.06)	86 (5.04)	72 (16.48)	1 (0.91)
**Employment**					
Employed full-time		939 (41.70)	565 (33.14)	279 (63.84)	95 (86.36)
Employed part-time		84 (3.73)	60 (3.52)	16 (3.66)	8 (7.27)
Unemployed		467 (20.74)	435 (25.51)	32 (7.32)	0 (0)
Student		463 (20.56)	372 (21.82)	88 (20.14)	3 (2.73)
Retired		229 (10.17)	207 (12.14)	19 (4.35)	3 (2.73)
Self-employed		70 (3.11)	66 (3.87)	3 (0.69)	1 (0.91)
**Marital status**					
Single		851 (37.79)	629 (36.89)	192 (43.94)	30 (27.27)
Married		1,281 (56.88)	983 (57.65)	220 (50.34)	78 (70.91)
Divorced		95 (4.22)	72 (4.22)	21 (4.81)	2 (1.82)
Widowed		25 (1.11)	21 (1.23)	4 (0.92)	0 (0)
**Income**					
<1331 USD		885 (39.30)	754 (44.22)	122 (27.92)	9 (8.18)
1,331–2,662 USD		504 (22.38)	355 (20.82)	88 (20.14)	61 (55.45)
2,663–5,325 USD		582 (25.84)	440 (25.81)	122 (27.92)	20 (18.18)
<5,325 USD		281 (12.48)	156 (9.15)	105 (24.03)	20 (18.18)
**Location**					
Middle regions		363 (16.12)	258 (15.13)	77 (17.62)	28 (25.45)
Western regions		1,451 (64.43)	1,082 (63.46)	308 (70.48)	61 (55.45)
Northern regions		111 (4.93)	89 (5.22)	16 (3.66)	6 (5.45)
Southern regions		123 (5.46)	96 (5.63)	17 (3.89)	10 (9.09)
Eastern regions		204 (9.06)	180 (10.56)	19 (4.35)	5 (4.55)
**Nationality**					
Saudi		2,022 (89.79)	1,542 (90.44)	373 (85.35)	107 (97.27)
Non-Saudi		230 (10.21)	163 (9.56)	64 (14.65)	3 (2.73)
Have you been diagnosed with Covid-19 disease?	Yes	48 (2.13)	33 (1.94)	9 (2.06)	6 (5.45)
	No	2,204 (97.87)	1,672 (98.06)	428 (97.94)	104 (94.55)
Has anyone in your family been diagnosed with Covid-19 disease?	Yes	79 (3.51)	57 (3.34)	17 (3.89)	5 (4.55)
	No	2,173 (96.49)	1,648 (96.66)	420 (96.11)	105 (95.45)
Have you had contact with any Covid-19 patients?	Yes	59 (2.62)	15 (0.88)	44 (10.07)	0 (0)
	No	2,193 (97.38)	1,690 (99.12)	393 (89.93)	110 (100)

*USD, United States dollar; COVID-19, Coronavirus disease 2019*.

**Figure 2:**
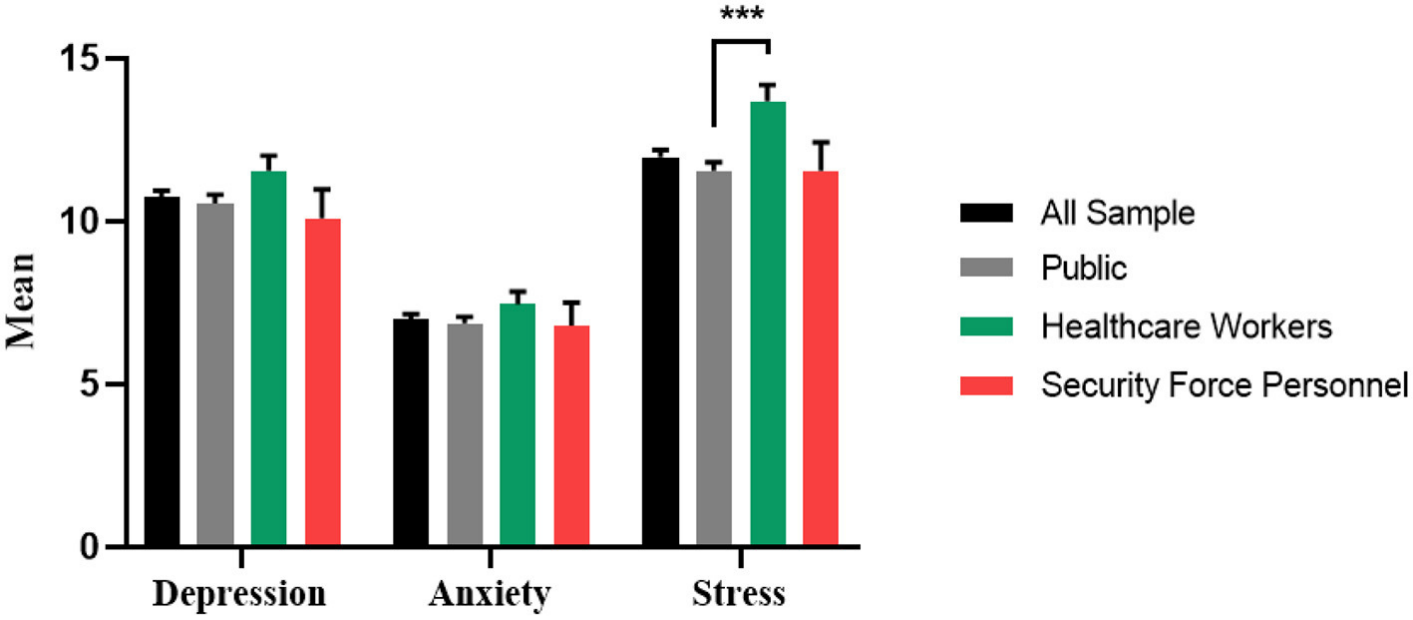
Mean depression, anxiety and stress scores: total sample, public, healthcare workers and security force personnel. ***P ≤ 0.001.

The means and standard deviations of the depression and anxiety DASS-21 scores for the whole sample (10.73 ± 10.29 and 6.98 ± 8.30, respectively) were in the range of mild depression and anxiety (Figure 2). In contrast, the means and standard deviations of the DASS-21 stress score were within the normal range (11.97 ± 10.80). The mean stress score was within the normal range for healthcare workers (13.70 ± 10.68), but was higher than the mean score of the public (11.56 ± 10.89; *P* = 0.0006). There were otherwise no significant differences between DASS-21 scores of the public, healthcare workers, and security force personnel.

**Figure 3:**
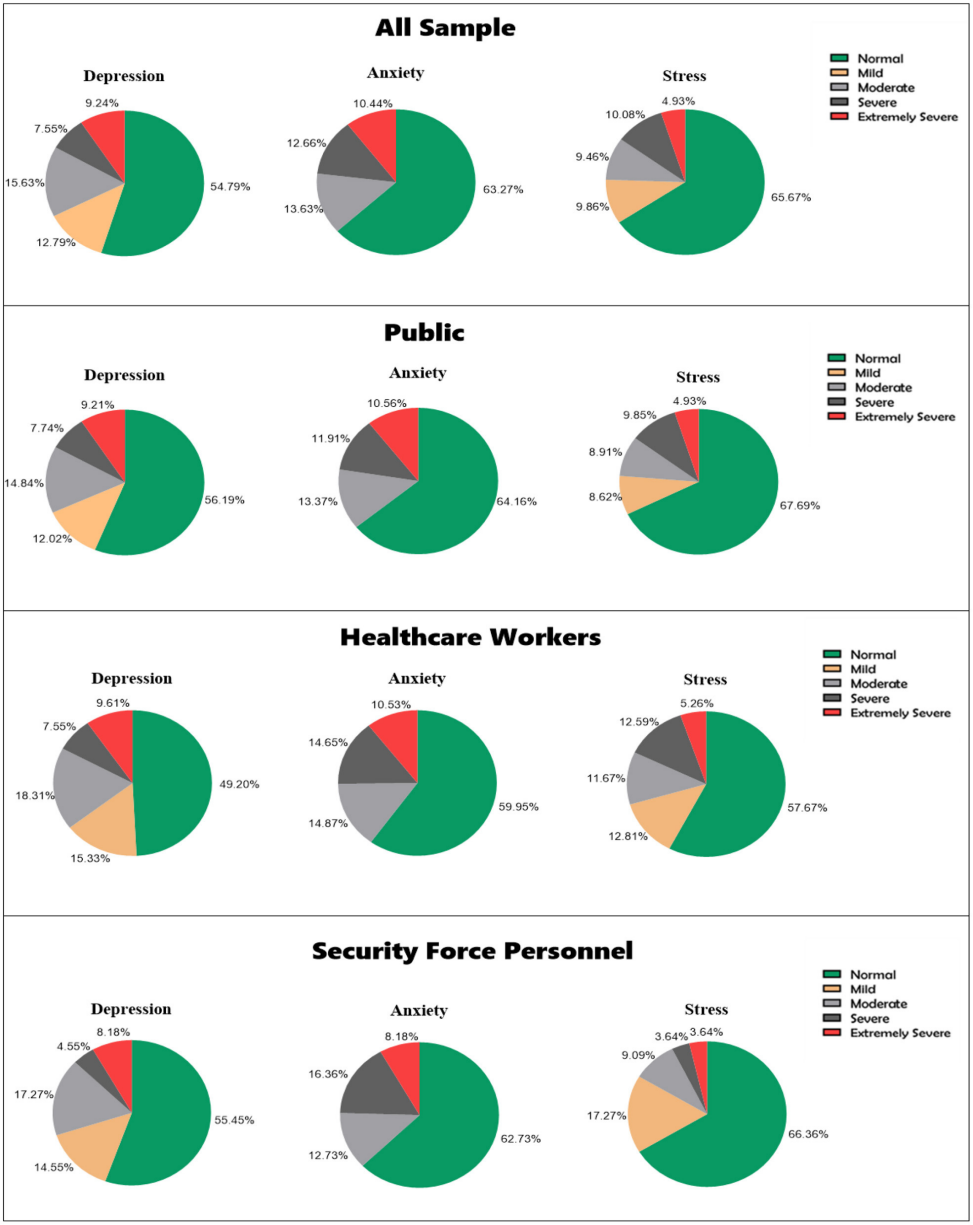
Proportion of participants with different levels of each psychological disorder: total sample.

Figure 3 shows the proportions of participants experiencing different levels of psychological distress as defined by the DASS-21. At least one-third of the population experienced one form of psychological distress. The proportion of healthcare workers who reported stress was significantly higher than that of the public or security personnel (*P* = 0.0004). Otherwise, there were no differences in the proportions of participants with depression or anxiety between the public, healthcare workers, and security personnel (*P* = 0.2109, *P* = 0.5662, respectively).

**Figure 4:**
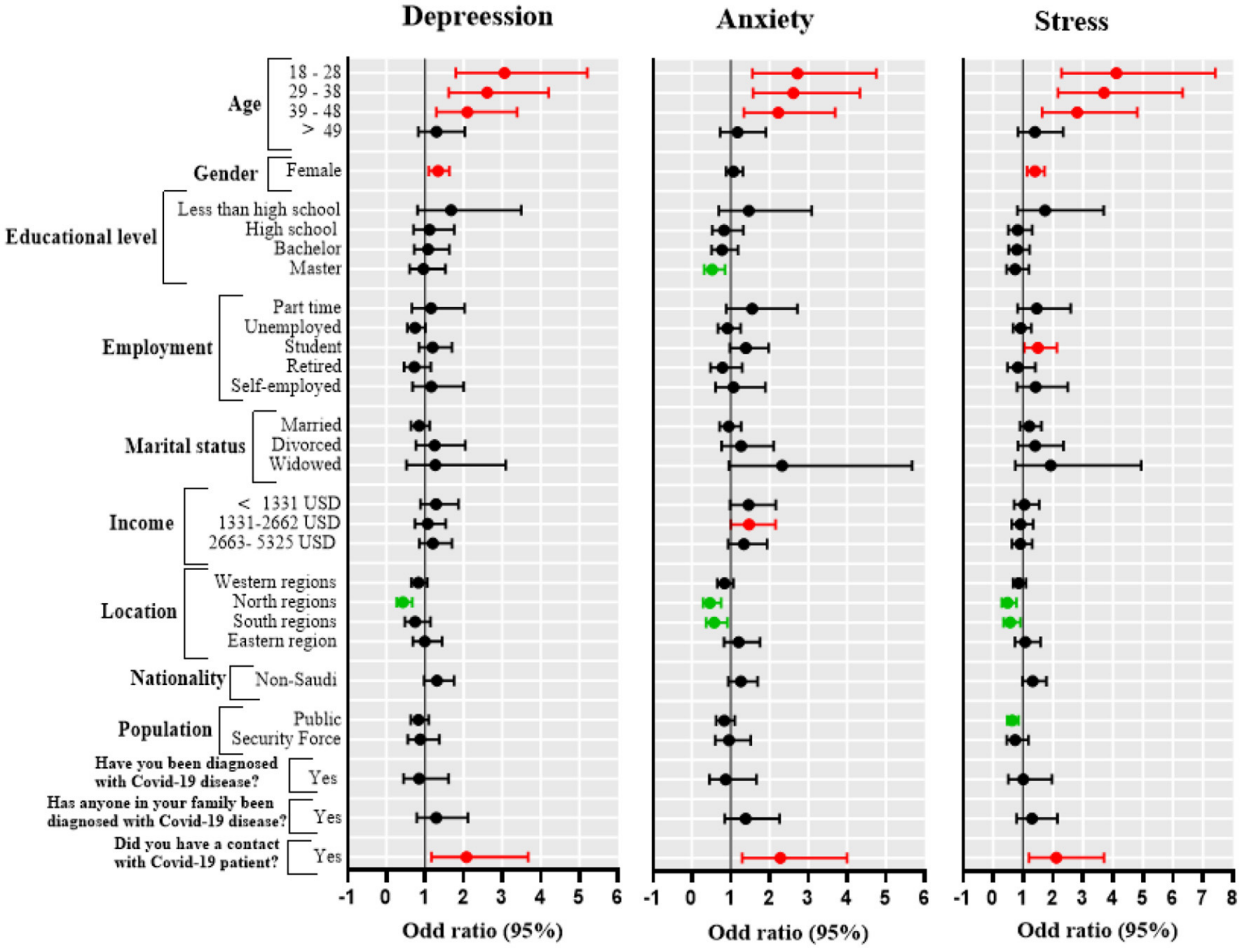
Forest plot of odds ratios and 95% confidence intervals (CI). Each panel represents the correlation between predictors and specific psychological parameters (depression, anxiety, stress). Each row represents a specific predictor with the corresponding odds ratio (dot) and 95% CI (horizontal line). The red horizontal line indicates a positive correlation; the green horizontal line indicates a negative correlation.

Using binary logistic regression analysis, we investigated the potential contribution of the independent sociodemographic variables of interest to the DASS-21 subscores (Figure 4). Women were more likely to have depression (OR = 1.34, 95% CI = 1.10–1.63, *P* = 0.0039) and stress (OR = 1.40, 95% CI = 1.14–1.72, *P* = 0.0015) than males. Additionally, participants aged ≤ 48 years were more likely to experience abnormal levels of depression, anxiety, and stress compared with participants aged ≥59 years (*P* < 0.05). Furthermore, significantly lower levels of depression (OR = 0.43, 95% CI = 0.27– 0.68, *P* = 0.0003), anxiety (OR = 0.46, 95% CI = 0.29– 0.75, *P* = 0.0017) and stress (OR = 0.48, 95% CI = 0.29– 0.78, *P* = 0.0033) were found in participants living in northern regions of the country compared with participants living in middle regions. The public was less likely to have abnormal levels of stress (OR = 0.64, 95% CI = 0.48–0.85, *P* = 0.0024) compared with healthcare workers. Education level, employment status, marital status, income, and nationality had no significant association with DASS-21 subscores. Furthermore, the results of the linear regression models were similar to the logistic regression models, except for the association of being a woman with depression subscore (see Supplementary Table 1).

## Discussion

This study reports the prevalence of psychological distress in the context of the COVID-19 pandemic and the strict curfew in the KSA. At the time of data collection, the number of COVID-19 cases was climbing exponentially and a nationwide, 24-h curfew had been in effect for an entire month. The literature contains much evidence that such circumstances during pandemics may result in a considerable burden of psychological distress. In addition to fears of infection and other health-related fears, economic hardship resulting from business and social activities restrictions may place a substantial burden on individuals and families (38, 39). Inevitable differences in perceptions of the effects of authoritative action during pandemics may contribute to increased anxiety or even public unrest. The results from this study show that one-third to one-half of subjects experienced significant levels of psychological distress, with about 10% of the population reporting the most severe level of psychological distress. Some sociodemographic characteristics appeared to be risk factors for higher psychological distress in our sample. Healthcare workers seemed to experience a higher percentage of psychological distress than the public, a finding that is not surprising given previous study findings (11, 39–43). Generally, younger and female subjects in our sample were more prone to psychological distress. This is similar to previous reports in the context of the COVID-19 pandemic (9, 36, 41). It is possible that the effects of sociocultural restrictions on daily life may be greater in young individuals than in older individuals.

In addition, young people may be more likely to follow the news on social media outlets (44). Consistent with our data, recent reports show that Chinese females have more symptoms of PTSD, depression, anxiety, and stress during COVID pandemics (9, 41, 45). Our results indicate that Master's graduates had significantly lower anxiety scores than Ph.D. graduates. Again, this is consistent with Chinese data collected during the COVID-19 pandemic (9). A possible explanation is that educated people are more health aware and tend to monitor their health more frequently (46). Contrary to our results, several studies have reported that low educational level is associated with greater psychological distress (47, 48). This may be because such individuals are 'blunters' of their health risks (i.e., they show avoidant behavior), suggesting a U-shaped association between education and psychological distress during pandemics that requires further study.

The present data are in line with data from other countries in the context of the pandemic and provide additional evidence of the mental health burden of the COVID-19 pandemic (9, 10, 12). However (and even though we did not perform a comparative study between countries), there is no evidence from the local Saudi data that the strict curfews and restrictions have led to more severe psychological distress in the KSA than in other countries. The Iranian (22) and Australian (12) populations showed higher DASS scores than Saudi. However, at the time of the Iranian and Australian data collections, there were restrictions on travel and mass gatherings and no strict complete curfews. Scores for Portuguese and Singapore samples were lower than our scores (18, 40), even though the Portuguese study data collection (on March 23) occurred four days after the Portuguese government had declared an emergency state and applied tight restrictions. In the Singapore study, Tan et al. acknowledged the limitations of conducting the study early in the outbreak, limiting the findings' generalizability (40). However, given the many sociocultural, health policy, political and demographic differences between these countries, firm conclusions about the effects of curfews and social restrictions cannot be drawn. Furthermore, the trends in COVID-19 cases and deaths are not homogeneous across countries. The KSA death rate has been one of the lowest in the world (49). The low death rate may be partly related to the strict policies applied in the country and may have indirectly balanced the potential increase in psychological distress caused by these policies. Another factor that may have balanced out the potential increase in anxiety resulting from tight restrictions is that the curfews and restrictions reduced the chance of individuals coming into contact with COVID-19 cases. Our data show that history of contact with COVID-19 patients was associated with a higher risk of anxiety, which is consistent with previous data showing that the prospect of coming into contact with infected cases during a pandemic significantly increases anxiety during these periods (9, 12, 39, 50).

During pandemics, risk communication with the public plays a crucial role in shaping the psychological response during these difficult times, especially in countries where strict curfews are applied (51). People are more likely to adhere to authority regulations if they believe that the authorities are transparent and provide sufficient clear information. The government of Saudi Arabia held daily press briefings organized and conducted by the Ministry of Health. An application was launched and made available to all citizens. The application allowed people to access data and graphs on pandemic trends and learn about the caseloads in the regions relevant to them (52). Different communication strategies were applied during this pandemic. A media campaign was launched to appeal to people's logical and emotional sides. The campaign portrayed adherence to health and curfew regulations as a patriotic act and a social responsibility that protects everybody. Evidence suggests that emotional approaches may have a stronger appeal than logical approaches (51, 53). Furthermore, the government instituted heavy fines on breaking curfews to discourage people from spreading the infection, which is another communication strategy that is helpful during pandemics (51).

This study has some limitations. First, this was not a comparative study with simultaneous prospective data collection from different countries; therefore, the data cannot be used to draw firm conclusions about the effect of curfew regulations on a psychological burden during pandemics. Second, we used a convenience sample. This may have resulted in selection bias: individuals with very low or very high anxiety levels may have refrained from participating in the study because they avoid accessing the news media on which the study tool was disseminated. Third, the design was sufficiently powerful to detect significant differences in psychological distress scores but may not have been sufficiently powerful to detect minor differences between some subgroups (such as older adults, who constituted a relatively small proportion of our sample). Fourth, the sample was primarily drawn from the western region of the KSA, limiting the generalizability of the findings to the rest of the country, let alone to other countries.

## Conclusions

To the best of our knowledge, this is the first survey from the KSA to demonstrate the psychological impact of the COVID-19 pandemic. It supplements existing Chinese data on the psychological effects of strict curfews and social restrictions. We showed that there is indeed a psychological burden resulting from the pandemic in the KSA, but that this does not appear to differ from that of other countries with less strict regulations. These findings could inform health policy and further studies to identify appropriate responses to global pandemics. For instance, public health interventions that ameliorate the risk perception of COVID-19 through the dissemination of adequate and targeted health information could be a successful measure to mitigate the psychological impact of the Covid-19 pandemic. Our data suggest that strict curfews and policy regulations are not necessarily associated with a more significant net psychological burden. However, further studies with purposeful sampling and pre-planned cross-country comparisons are needed.

## Data Availability Statement

The raw data supporting the conclusions of this article will be made available by the authors, without undue reservation.

## Ethics Statement

The studies involving human participants were reviewed and approved by the Research Ethics Committee of King Abdulaziz University. The patients/participants provided their written informed consent to participate in this study.

## Author Contributions

BA, YA, FA, and HT designed the study, supervised the work and wrote the manuscript. HA and AB collected the data. YA conducted the statistical analysis. All authors contributed to the article and approved the submitted version.

## Conflict of Interest

The authors declare that the research was conducted in the absence of any commercial or financial relationships that could be construed as a potential conflict of interest.

## Publisher's Note

All claims expressed in this article are solely those of the authors and do not necessarily represent those of their affiliated organizations, or those of the publisher, the editors and the reviewers. Any product that may be evaluated in this article, or claim that may be made by its manufacturer, is not guaranteed or endorsed by the publisher.
