# Supplementary material for: Psychological Distress During COVID-19 Curfews and Social Distancing in Saudi Arabia: A Cross-Sectional Study
Source: Front Public Health. 2022 Jan 4;9:792533. doi: 10.3389/fpubh.2021.792533 (PMC8764301; doi:10.3389/fpubh.2021.792533)
Supplement: Supplementary file 1 [file Table_1.DOCX]

Supplementary Material

**Supplementary Table 1:** Association between DASS-21 and sociodemographic characteristics of the study sample.

|  | Depression | | Anxiety | | Stress | |
| --- | --- | --- | --- | --- | --- | --- |
|  | B (95% CI) | p-value | B (95% CI) | p-value | B (95% CI) | p-value |
| Age | |  |  |  |  |  |
| 18 - 28 | 6.33 (2.20-10.45) | 0.013 | 4.79 (1.42-8.17) | 0.0054 | 8.54 (4.21-12.88) | 0.0001 |
| 29 - 38 | 5.75 (1.80-9.70) | 0.0027 | 3.98 (0.75-7.21) | 0.0157 | 7.78 (3.63-11.93) | 0.0002 |
| 39 - 48 | 4.87 (0.92-8.82) | 0.0043 | 3.34 (0.11-6.57) | 0.0429 | 6.73 (2.58-10.89) | 0.0015 |
| 49 - 58 | 2.75 (-1.09- 6.58) | 0.0158 | 1.80 (-1.34-4.94) | 0.2602 | 3.44 (-0.59-7.47) | 0.094 |
| 59 - 68 | 2.32 (-1.59-6.23) | 0.1602 | 1.65 (-1.54-4.85) | 0.3101 | 2.41 (-1.69-6.52) | 0.2496 |
| > 68 | REF |  | REF |  | REF |  |
| Gender | |  |  |  |  |  |
| Female | 1.18 (0.25-2.11) | 0.0125 | 0.48 (-0.28-1.24) | 0.2192 | 1.60 (0.62-2.57) | 0.0014 |
| Male | REF |  | REF |  |  |  |
| Education | |  |  |  |  |  |
| Bachelor’s degree | 0.77 (-1.122.67) | 0.4239 | 0.12 (-1.43-1.68) | 0.875 | -0.81 (-2.81-1.18) | 0.4252 |
| High school degree | 0.74 (-1.37- 2.86) | 0.4908 | 0.46 (-1.27-2.20) | 0.6004 | -0.53 (-2.75-1.70) | 0.6428 |
| Less than high school | 1.81 (-1.62- 5.25) | 0.3009 | 2.82 (0.01-5.64) | 0.0491 | 1.04 (-2.58-4.66) | 0.5726 |
